# Synthesis of Unusually High Valent Perovskite Oxide from the Highly Oxidized Coprecipitation Precursor

**DOI:** 10.1021/jacs.6c04051

**Published:** 2026-06-18

**Authors:** Takumi Nishikubo, Ryan J. Paull, Takatoshi Hirooka, Kana Matsuno, Koki Maebayashi, Jiong Ding, Hidetaka Kasai, Shigeo Mori, Takafumi Yamamoto, Kenneth R. Poeppelmeier, Masaki Azuma

**Affiliations:** † 34762 Kanagawa Institute of Industrial Science and Technology, 705-1 Shimoimaizumi, Ebina, Kanagawa 243-0435, Japan; ‡ Research Center for Autonomous Systems Materialogy (ASMat), Institute of Integrated Research, Institute of Science Tokyo, 4259 Nagatsuta-cho, Midori-ku, Yokohama, Kanagawa 226-8501, Japan; § Materials and Structures Laboratory, Institute of Integrated Research, Institute of Science Tokyo, 4259 Nagatsuta-cho, Midori-ku, Yokohama, Kanagawa 226-8501, Japan; ∥ Department of Materials Science, Graduate School of Engineering, 12936 Osaka Metropolitan University, 1-1, Gakuen-cho, Naka-ku, Sakai, Osaka 599-8531, Japan; ⊥ Department of Chemistry, Graduate School of Science, 12918 Kyoto University, Kitashirakawa Oiwake-cho, Sakyo, Kyoto 606-8502, Japan; # Department of Chemistry, 3270 Northwestern University, 2145 Sheridan Rd. Evanston, Illinois 60208-3113, United States

## Abstract

Perovskite oxides containing cations with unusually high-valent states such as Fe^4+^, Ni^3+^, and Cu^3+^ have attracted significant attention, often requiring a strong oxidizing atmosphere. This study addresses the challenges associated with the conventional precursor preparation for the high-pressure synthesis of BiNi_1–*x*_Fe_*x*_O_3_, a material exhibiting negative thermal expansion (NTE), specifically the need for mixed oxidants and the emission of NO_*x*_. We successfully established a modified method for preparing a highly oxidized, amorphous precursor containing Bi^5+^ and Ni^3+^ ions, ensuring high elemental dispersion by employing a reverse coprecipitation method with simultaneous oxidation using hypochlorite ions, enabling the synthesis of the target phase without the addition of external oxidizing agents. When subjected to high-pressure and high-temperature treatment, the BiNi_1–*x*_Fe_*x*_O_3_ phase crystallized directly from the amorphous precursor at a relatively low temperature (750 °C) and in a short time of less than 1 min, unlike the crystalline precursor prepared by conventional methods. Furthermore, leveraging the advantage of direct crystallization from the amorphous phase, we demonstrated that reducing the heating duration allows for the fabrication of fine particles, decreasing the size from 15 to 5 μm. These fine particles exhibited NTE over a wider temperature range without any degradation of the volume shrinkage magnitude. This synthetic method provides a safe, pollution-free, and potentially scalable route for BiNi_1–*x*_Fe_*x*_O_3_ fine particles and is expected to be applicable to the synthesis of other oxides containing anomalously high-valent ions.

## Graphical abstract

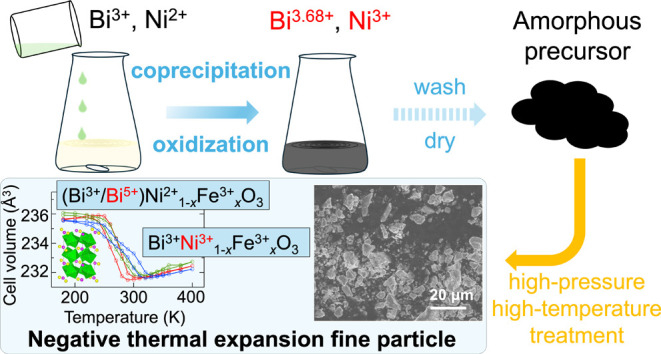

## Introduction

Transition metal oxides containing anomalous valence ions are attracting significant research interest due to their diverse physical properties, such as superconductivity, ferroelectricity, and giant magnetoresistance. Synthesis methods for such oxides vary widely, including high oxygen pressure and ozone oxidation methods, leading to the discovery of new oxides and the exploration of novel physical properties.^1,2^ In recent decades, perovskite oxides comprising unusually high-valent cations such as Fe^4+^, Ni^3+^, and Cu^3+^ have been obtained by high-pressure (HP) synthesis at several GPa.^3–12^ In order to stabilize such high-valent cations, it is necessary to generate a very strong oxidation atmosphere by using oxidants that release oxygen at high temperatures (HTs) together with the starting materials. In this study, we focused on a negative thermal expansion material BiNi_1–*x*_Fe_*x*_O_3_, which is synthesized under high-oxygen-pressure and high-temperature condition generated by the decomposition of KClO_4_ at 6 GPa and 1000 °C.^13,14^ We successfully synthesized this compound from a highly oxidized amorphous precursor without using an additional oxidizing agent.

The parent compound, BiNiO_3_, has a characteristic valence state of Bi^3+^_0.5_Bi^5+^_0.5_Ni^2+^O_3_ at ambient pressure, in which two bismuth sites disproportionated into 3+ and 5+ are columnarly ordered in a triclinic √2*a* × √2*a* × 2*a* unit cell, where *a* is the lattice parameter of a cubic perovskite.^3^ Applying pressure to this compound causes an intermetallic charge transfer between Bi^5+^ and Ni^2+^ resulting in an orthorhombic Bi^3+^Ni^3+^O_3_ high-pressure phase. Because of the shrinkage of the NiO_6_ octahedra, which constitute the perovskite framework, 2.6% volume shrinkage is observed.^15^ Fe^3+^ substitution for Ni^2+^ enables the charge transfer transition to occur by heating at ambient pressure, leading to industrially important negative thermal expansion (NTE).^13,14^ This transition is of first order, but the coexistence of a large volume low-temperature phase and a small volume low-temperature phase with varying fractions as a function of temperature results in a linear decrease in the weighted average unit cell volume. It has been shown that dispersing only 18 vol % of this compound as a filler in the bisphenol A epoxy resin can suppress the large thermal expansion (∼80 ppm/K) of the resin, and a zero-thermal-expansion composite is obtained.^13^

BiNi_1–*x*_Fe_*x*_O_3_ is attracting attention as a material for thermal expansion control in various areas where precise positioning is required, but the process of preparing the precursor causes problems in addition to the requirement of the HP condition of 6 GPa. In previous studies, the precursors of BiNi_1–*x*_Fe_*x*_O_3_ were prepared by the evaporation and thermal decomposition of metal nitric acid solutions of Bi, Ni, and Fe, which emitted large amounts of nitrogen oxides, making industrial production difficult from a viewpoint of pollution.^3,13,14^ In addition, since the synthesized precursor contains trivalent Bi and divalent Ni compounds, mixing of oxidants, which can be as much as 1/3 of the volume and involves the risk of blowout during synthesis, requires washing after synthesis. In this study, we established a preparation process to make a highly oxidized amorphous precursor that ensures high elemental dispersion and eliminates the necessity of adding oxidizing agents postsynthesis. The gel obtained by the inverse coprecipitation method can be dehydrated and crystallized by low-temperature heating, and the particle size and shape can be controlled by adjusting the reaction temperature and time.^16^ So, we employed a reverse coprecipitation method for atomic dispersion and obtained highly oxidized amorphous precursors by simultaneous oxidation with hypochlorite ions. This method allows us to obtain the target phase under mild conditions due to the gain in lattice energy and solves the above-mentioned problems. The target compound, BiNi_1–*x*_Fe_*x*_O_3_, was obtained by HP-HT treatment of such a prepared precursor without mixing oxidants. This process does not emit nitrogen oxides, is safe, and, furthermore, can be scaled up. It is also clarified that the synthesis by direct crystallization from the amorphous precursor in a short heating time enables the production of fine particles.

## Experimental Section

### Synthesis

First, we established a precursor preparation method for high-pressure synthesis. An acidic solution of bismuth, nickel, and iron nitrates was used for the reverse coprecipitation method. Bismuth nitrate (Bi­(NO_3_)_3_·5H_2_O), nickel nitrate (Ni­(NO_3_)_2_·6H_2_O), and iron nitrate (Fe­(NO_3_)_3_·9H_2_O) with stoichiometric metal ratios were dissolved in dilute nitric acid, and a green solution was obtained. Coprecipitation and oxidation were carried out simultaneously by dropping the above-mentioned aqueous solution into an aqueous sodium hydroxide (NaOH)/sodium hypochlorite (NaClO) mixture to obtain (ox)­hydroxide gels. The precipitate obtained in this way was separated by centrifugation, collected, and dried to yield the reaction precursor. Using this precursor, BiNi_1–*x*_Fe_*x*_O_3_ was synthesized under HP and HT condition. The precursors were packed into Au containers, put into pyrophyllite cubes, then treated at 4–6 GPa and 1023–1273 K for 30 min in a cubic anvil-type high-pressure apparatus.

### Characterization

The laboratory powder X-ray diffraction (XRD) patterns for phase identifications were obtained with a diffractometer (D8 Advance, Bruker) equipped with a Cu Kα radiation source. High angle annular dark field scanning transmitting electron microscopy (HAADF-STEM) and selected area electron diffraction (SAED) were carried out using a transmission electron microscope (JEM-ARM200F, JEOL Co., Ltd., Japan) to confirm the amorphous state. For structural refinement, the synchrotron X-ray diffraction (SXRD) patterns were collected with a large Debye–Scherer camera equipped with MYTHEN detectors installed at the BL02B2 beamline of SPring-8.^17^ The samples were sealed into Lindemann glass capillaries with an inner diameter of 0.1 mm. The wavelength was calibrated from Rietveld analysis of a diffraction pattern of CeO_2_ and was precisely λ = 0.420568 Å, 0.420118 Å, or 0.420624 Å. The temperature of the samples was changed by flowing nitrogen gas with a heating rate of 20 K/min and 30 s waiting time before the measurements. To calculate the average lattice volume, the lattice parameters and fraction of each phase were refined by Rietveld analysis for the SXRD patterns using TOPAS software. Structural parameters of the low-temperature and high-temperature phases were refined by RIETAN-FP.^18^ To further investigate the formation of BiNi_0.85_Fe_0.15_O_3_ from the amorphous precursor by HP-HT treatment, in situ observation of the reaction process was performed by energy dispersive synchrotron X-ray powder diffraction using a cubic anvil-type high-pressure apparatus SMAP2 installed at BL14B1 of SPring-8.^19^ White beam X-ray from a bending magnet source was injected into the samples through the HP cell and was detected with a Ge solid-state detector fixed at 2θ = 4.3°. The XRD patterns were taken at various pressures and temperatures. For comparison, we also observed the reaction of a conventional precursor obtained by evaporation-drying solidification of nitrates. To evaluate the valences of Bi and Ni, soft X-ray absorption spectroscopy for Ni L edge and O K edge was performed at BL27SU of SPring-8.^20^ Particle shape and size were examined using a field-emission scanning electron microscope (FE-SEM: Hitachi High-Tech S-4800) with an acceleration voltage of 15 kV.

## Results and Discussion

As mentioned in the experimental section, we established a precursor preparation including a high-valent Ni^3+^ ion. This method is based on the reverse coprecipitation method with oxidization simultaneously occurring. Nickel precipitates out as nickel­(II) hydroxide (eq 1), but it is simultaneously oxidized by sodium hypochlorite as nickel oxyhydroxide (eq 2). The reaction formula follows.

1 $${Ni}^{2+}{+2OH}^{-}\to {Ni\left(OH\right)}_{2}$$

$${Ni}^{2+}{\left(OH\right)}_{2}+1/2NaClO$$

2 $${\to Ni}^{3+}O\left(OH\right)+1/2NaCl+1/2H_{2}O$$

The Bi^3+^ ion can be oxidized to Bi^5+^ by potassium ferricyanide with *E*_0_ = 0.43 eV.^21^ Thus, bismuth is also considered to be partially oxidized to the pentavalent by-state sodium hypochlorite (eqs 3–5)). TG measurements revealed a gradual weight loss up to 600 K, attributed to water desorption. The weight remains constant up to 840 K and then begins to decrease again. During this decrease, oxygen is released, while the precursor crystallizes into a mixture of Bi_25_FeO_39_, NiO, and Fe_2_O_3_.(Figure 1a) Estimating the oxygen content of the precursor from this decrease yields BiNi_0.85_Fe_0.15_O_3.34_. Bi is then 3.68 valent if all Ni is trivalent. Thus, this precursor is oxygen-rich.

3 $${Bi}^{3+}{+3OH}^{-}\to {Bi\left(OH\right)}_{3}$$

4 $$2{Bi\left(OH\right)}_{3}\to {Bi}_{2}O_{3}+H_{2}O$$

5 $${Bi}_{2}^{3+}O_{3}+2NaClO\to {Bi}_{2}^{5+}O_{5}+2NaCl$$

**Figure 1:**
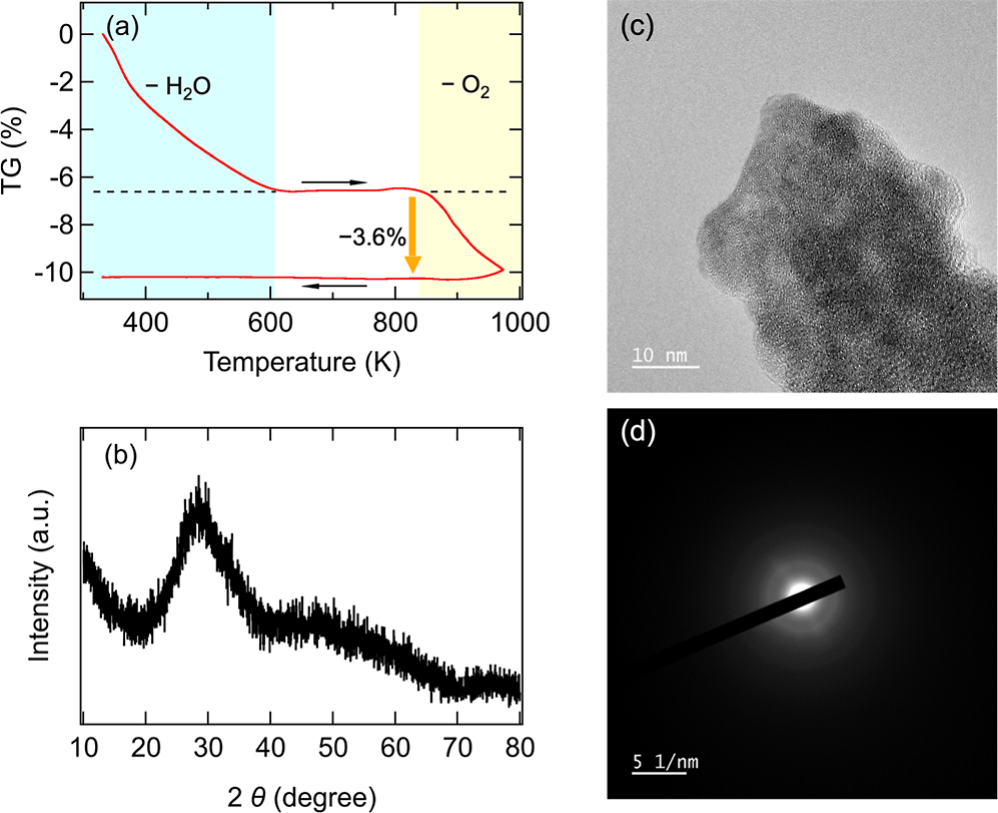
(a) Water and oxygen absorption observed by thermogravimetric, (b) laboratory X-ray diffraction pattern, (c) HAADF-STEM image, and (d) SAED pattern of precursor for BiNi_0.85_Fe_0.15_O_3_, revealing the amorphous nature.

The light-green Bi- and Ni-containing acidic solution produced a black precipitate through these coprecipitation and oxidation reactions (Supporting Information Movie S1). The precipitate obtained in this way was separated by centrifugation, collected, and dried to yield the reaction precursor, which was confirmed to be amorphous from the X-ray diffraction pattern, STEM image, and SAED pattern (Figure 1b–d).

Since the precursor is amorphous, it is impossible to estimate the valence of cations from X-ray diffraction. We evaluated the valences of Bi and Ni by soft X-ray absorption spectroscopy at BL27SU of SPring-8.^20^ We found a pre-edge peak at the lower-energy side (529 eV) of the edge around 532 eV in the O K edge (Figure 2a). This is also seen in BiNiO_3_, suggesting that it is derived from Bi^5+^ (Bi^3+^ *L*_2_: *L* denotes ligand hole). Similar pre-edge peak features that can be attributed to oxygen holes have been observed in BaBiO_3_ and Ba_1–*x*_K_*x*_BiO_3_.^22,23^ Also, the Ni L-edge (Figure 2b) shows a spectrum different from that of Ni^2+^O, and the spectrum of the precursor is similar to that of Ni^3+^OOH.^24^ Based on the above results, it is expected that BiNi_1–*x*_Fe_*x*_O_3_ can be synthesized without the addition of oxidants because the amorphous precursor contains both Bi^5+^ and Ni^3+^.

**Figure 2:**
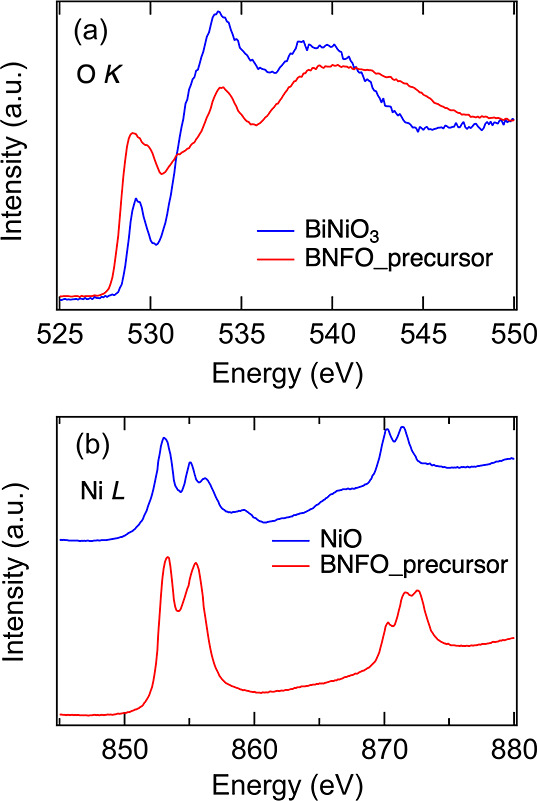
Soft X-ray absorption spectra of amorphous precursor for BiNi_0.85_Fe_0.15_O_3_. (a) O K edge with BiNiO_3_ reference and (b) Ni L edge with NiO reference.

Next, we synthesized BiNi_1–*x*_Fe_*x*_O_3_ by HP-HT treatment directly from the precursor obtained using sodium hypochlorite. The perovskite compound with a single triclinic phase was obtained for *x* = 0.05, and coexisting triclinic and orthorhombic phases were obtained for *x* = 0.1 and 0.15, as shown in Figure 3a. For each composition, the phase transition from triclinic to orthorhombic is observed on heating, indicating a negative thermal expansion. As in the previous report of BiNi_1–*x*_Fe_*x*_O_3_, the transition temperature decreases with increasing *x* (Figure 3b), indicating that the operating temperature of thermal expansion can be controlled by the *x* value. Results of Rietveld analysis for the low- and high-temperature phases of the *x* = 0.15 sample are shown in Figure 3c,d with refined crystallographic parameters summarized in Table 1.

**Figure 3:**
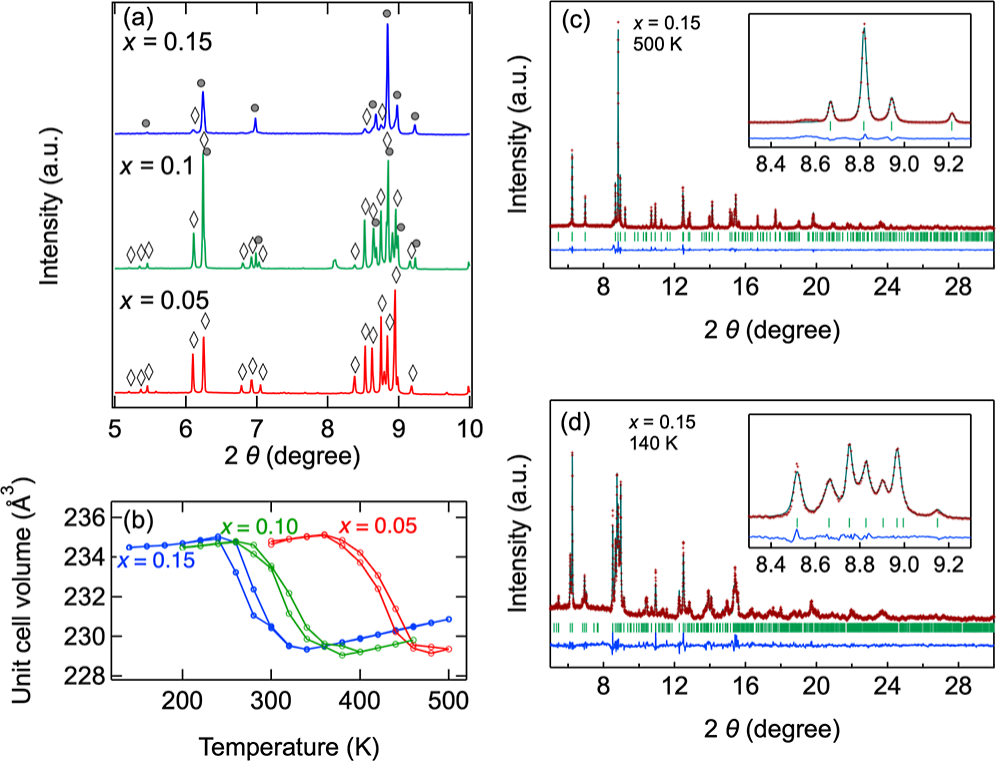
NTE behaviors of BiNi_1–*x*_Fe_*x*_O_3_ (*x* = 0.05, 0.10, and 0.15) synthesized from a highly oxidized coprecipitated precursor. Synchrotron X-ray diffraction patterns with a wavelength of 0.413854 Å (a), Temperature dependence of weighed average unit cell volume (b), and Rietveld analysis results of *x* = 0.15 samples at 500 K (c) and 140 K (d). White diamond marks and gray circles in (a) denote the LT triclinic and HT orthorhombic phases, respectively.

**Table 1:** Refined Crystallographic Parameters of Low- and High-Temperature Phases (140 and 500 K) of the *x* = 0.15 Sample

140 K						
triclinic phase, space group *P-*1						
atoms	positions	*g*	*x*	*y*	*z*	*B*_iso_ (Å^2^)
Bi1	2*i*	1	0.0072(7)	0.0456(6)	0.2385(3)	0.21(4)
Bi2	2*i*	1	0.5099(7)	0.4384(6)	0.7321(4)	
Ni1/Fe1	1*d*	0.85/0.15	0.5	0	0	0.13(7)
Ni2/Fe2	1*c*	0.85/0.15	0	0.5	0	
Ni3/Fe3	1*f*	0.85/0.15	0.5	0	0.5	
Ni4/Fe4	1*g*	0.85/0.15	0	0.5	0.5	
O1	2*i*	1	–0.0800(9)	0.4758(2)	0.2744(9)	0.39(9)
O2	2*i*	1	0.4023(2)	0.0369(2)	0.7661(18)	
O3	2*i*	1	0.8531	0.1643	–0.0442	
O4	2*i*	1	0.3165	0.3370	0.0383	
O5	2*i*	1	0.2312	0.7906	0.4720	
O6	2*i*	1	0.6862	0.6728	0.5306	
*Z* = 4, *a* = 5.3724(5) Å, *b* = 5.6573(5) Å, *c* = 7.7191(8) Å, *α* = 91.471(3)°, *β* = 89.911(3)°, *γ* = 91.092(3)°, *V* = 234.487(19) Å^3^						
*R*_B_ = 3.426%*, R*_wp_ = 9.937%, *R*_p_ = 7.819%, *R*_e_ = 7.542%, *S* = 1.3176						

500 K						
orthorhombic phase, space group *Pbnm*						
atoms	positions	*g*	*x*	*y*	*z*	*B*_iso_ (Å^2^)
Bi	4*c*	1	–0.0048(5)	0.0513(5)	0.25	1.14(2)
Ni/Fe	4*b*	0.85/0.15	0.5	0	0	0.57(4)
O1	4*c*	1	0.0859(3)	0.4695(2)	0.25	1.47(4)
O2	8*d*	1	0.6763(2)	0.3056(2)	0.0293(2)	
*Z* = 4, *a* = 5.38612(9) Å, *b* = 5.55681(4) Å, *c* = 7.71379(13) Å, *V* = 230.871(7) Å^3^						
*R*_B_ = 4.467%, *R*_wp_ = 9.171%, *R*_p_ = 6.793%, *R*_e_ = 7.833%, *S* = 1.1709						

In order to observe the synthesis route of BiNi_1–*x*_Fe_*x*_O_3_, synchrotron XRD under high-pressure and high-temperature condition using a white beam was performed. Figure 4a shows the reaction of the conventional precursor comprising Bi_25_FeO_39_, NiO, and Bi_2_O_3_ at 6 GPa. The reaction gradually progressed through two or more intermediate phases and completed at 950 °C. On the other hand, the peaks of the perovskite phase directly appeared in the case of the amorphous precursor prepared using the present process (Figure 4b), and the crystallization was completed at 750 °C. It should be noted that two peaks at X-ray energies of 77.108 and 74.815 keV correspond to the Bi Kα_1_ and Kα_2_ characteristic X-rays that remain unchanged with pressure, respectively. These indicate that, as expected, BiNi_1–*x*_Fe_*x*_O_3_ formed directly from the amorphous precursor at relatively low temperatures and in a short time without the need for an oxidant.

**Figure 4:**
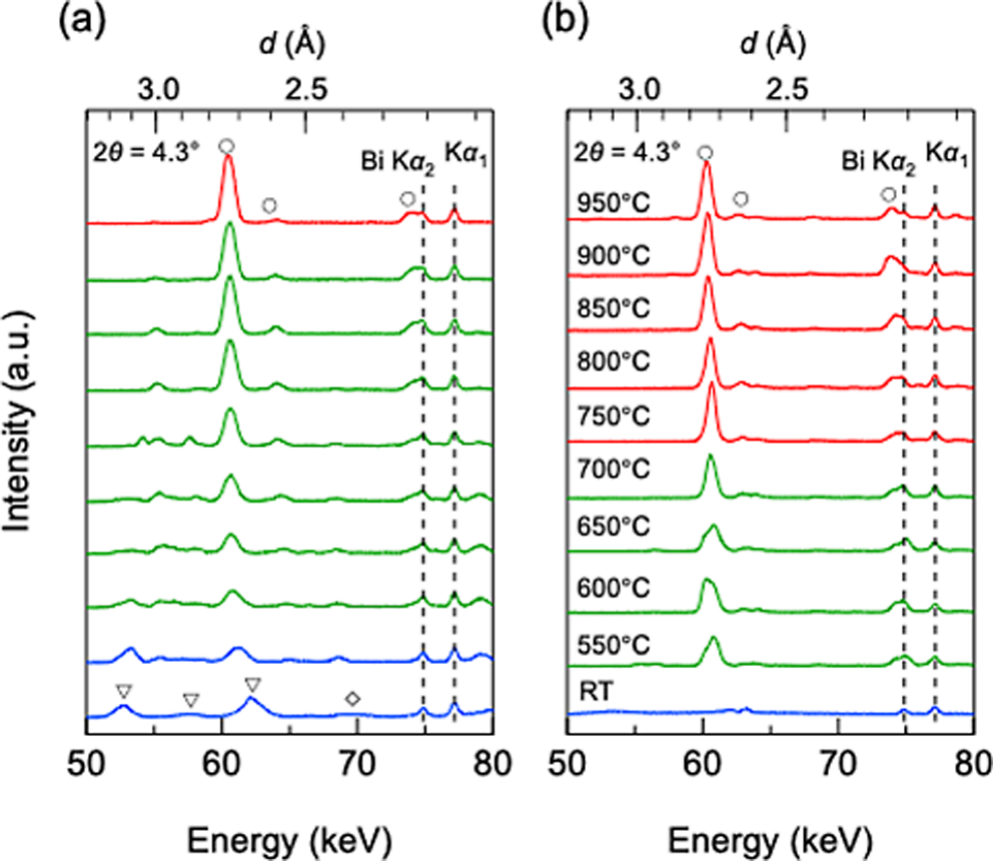
In situ X-ray diffraction experiments under high pressure for reactions of the precursor obtained by the conventional method with potassium perchlorate (a) and directly from the precursor obtained using sodium hypochlorite (b). Circle, triangle, diamond marks, and dashed line denote BiNi_0.85_Fe_0.15_O_3_ (orthorhombic), Bi_25_FeO_39_, NiO, and Bi Kα line.

Ceramic materials are generally obtained by solid-phase reactions between some crystallized phases, in which several raw materials are mixed and are reacted at high temperatures. Since the solid-phase reaction method requires thermal energy for elemental diffusion at the interface between the reactant particles and the intermediate products generated in the reaction process, a long heating time and intermediate crushing and mixing are necessary to obtain the desired phase. Grain growth occurs in this process. In the precursor obtained in the present study, the target phase forms directly from the amorphous phase, and the elements are highly dispersed. Therefore, we attempted to obtain fine particles by heating the precursor for a short time at an HP condition. The precursors charged in gold capsules were compressed to 4 GPa, and the temperature was increased to 1073 K in 5 min, held for 30, 15, or 0 min, and quenched before the pressure was released. Observation of the particles by scanning electron microscopy revealed that the size of the particles decreased from 15 μm in the 30 min sample to 5 μm in the 0 min sample (Figure 5a–d). The particle size profile is shown in Figure 5e-g shows the SXRD patterns of these samples at RT. Perovskite BiNi_0.85_Fe_0.15_O_3_ formed in all samples; note, however, that the fractions of LT triclinic and HT orthorhombic phases are different depending on the heating duration. Rietveld analyses of the synchrotron X-ray diffraction patterns at various temperatures confirmed the occurrence of NTE in all the samples, as shown in Figure 5h. The coefficient of linear thermal expansion of the 30 min sample is −158 ppm/K, but the phase transition became gradual as the heating time was decreased, reaching −76.8 ppm/K for the 5 min sample and −66.6 ppm/K for the 0 min sample. The magnitude of the volume shrinkage remains unchanged, indicating that the obtained particulate materials exhibited NTE over a wider temperature range without the degradation. Mechanical pulverization sometimes causes degradation of ceramics and suppresses NTE.^25^ However, the particles obtained through this method do not exhibit such deterioration, indicating that this process can effectively modify the ceramics without compromising their integrity. This method of synthesizing fine particles takes advantage of direct crystallization from amorphous precursors.

**Figure 5:**
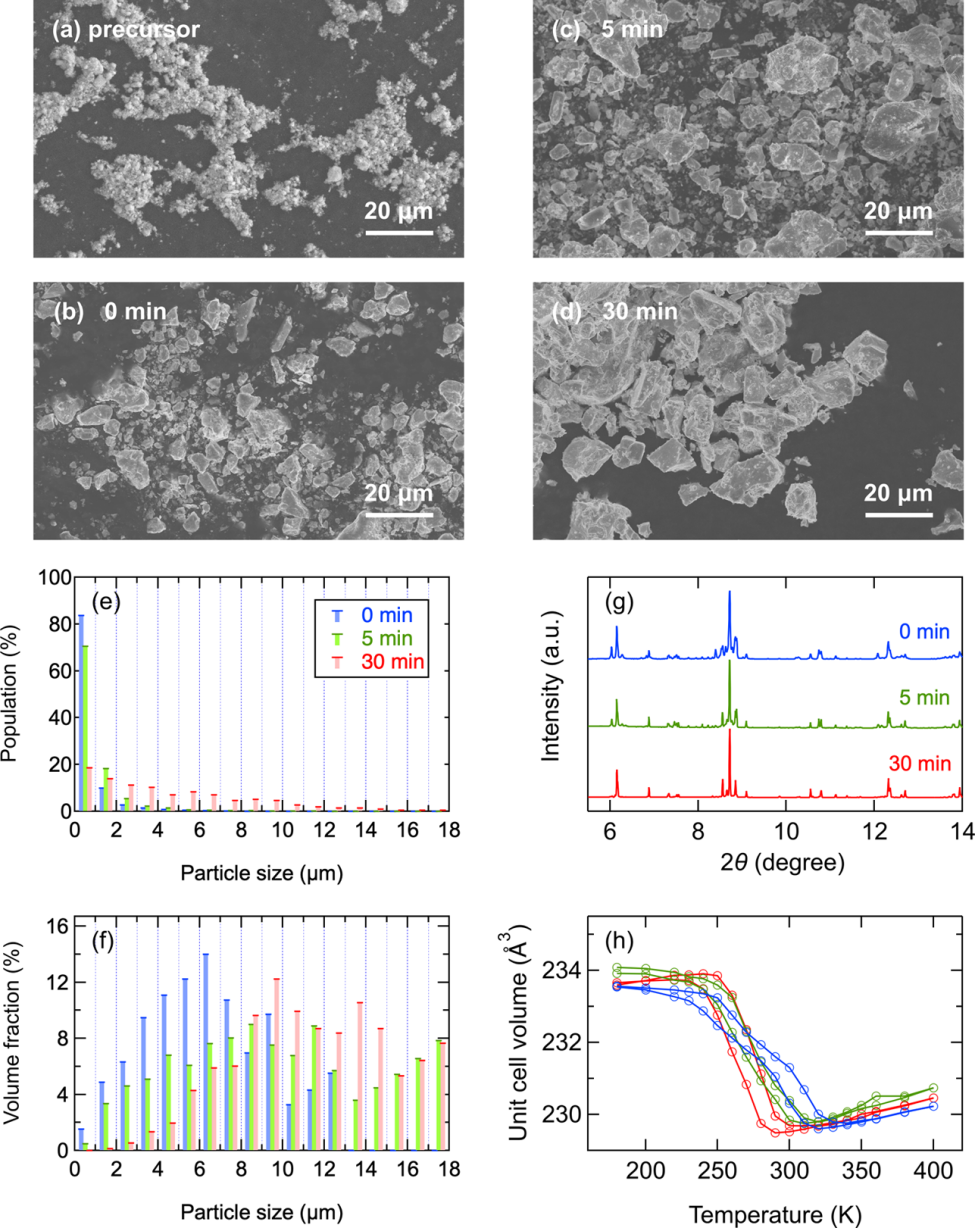
BNFO synthesized by varying the heating time. SEM images of precursor (a) and samples heated for 0 min (b), 5 min (c), and 30 min (d), population (e) and volume fraction (f) for each particle size, SXRD patterns on room temperature (g) and NTE properties (h) of the obtained powder samples. Blue: 0 min, green: 5 min and red: 30 min.

## Conclusion

We investigated the preparation of the precursor for the HP synthesis of BiNi_1–*x*_Fe_*x*_O_3_ containing unusually high-valent cations. We found that an amorphous precursor containing Bi^5+^ and Ni^3+^ ions can be obtained by coprecipitation by oxidation from aqueous solutions of nitrate dropped into a mixture of sodium hydroxide and sodium hypochlorite. Furthermore, the target phase was directly formed by heating this amorphous precursor under high pressure, and fine particles were successfully fabricated by heating the precursor for a short time. We demonstrate making a precursor for a batch reaction, but since this process allows for the production of slurry via a continuous reaction, it is believed that stable mass production is possible, provided that the concentration and flow rate are properly adjusted. In addition to the expected scale-up of the synthesis of the obtained negative thermal expansion fine particles, this synthetic method can be applied for the synthesis of Cu^3+^-containing YBa_2_Cu_3_O_7_ for a short time without annealing (see the Supporting Information). Thus, this method is expected to be utilized for the synthesis of other oxides containing anomalously high-valence ions.

## Supplementary Material





## Notes

Simultaneous coprecipitation and oxidation by dropping Bi, Ni, and Fe nitric acid solution into an aqueous sodium hydroxide (NaOH)/sodium hypochlorite (NaClO) mixture (MOV)
Synthesis of Cu^3+^ containing YBa_2_Cu_3_O_7_ from a highly oxidized coprecipitation precursor (PDF)
