# Supplementary material for: Synthesis of Unusually High Valent Perovskite Oxide from the Highly Oxidized Coprecipitation Precursor
Source: J Am Chem Soc. 2026 Jun 18;148(25):25898–904. doi: 10.1021/jacs.6c04051 (PMC13339637; doi:10.1021/jacs.6c04051)
Supplement: Supplementary file 2 [file ja6c04051_si_002.pdf]

Supporting Information for Publication

Synthesis of unusually high valent perovskite oxide from highly oxidized co-precipitation precursor

Takumi Nishikubo[1,2,3]*, Ryan J. Paull[4], Takatoshi Hirooka[3], Kana Matsuno[3], Koki Maebayashi[3], Jiong Ding[5], Hidetaka Kasai[5], Shigeo Mori[5], Takafumi Yamamoto[3,6], Kenneth R. Poeppelmeier[4] and Masaki Azuma[2,3,1]

[1] Kanagawa Institute of Industrial Science and Technology, 705-1 Shimoimaizumi, Ebina, Kanagawa 243-0435, Japan

[2] Research Center for Autonomous Systems Materialogy (ASMat), Institute of Integrated Research, Institute of Science Tokyo, 4259 Nagatsuta-cho, Midori-ku, Yokohama, Kanagawa 226-8501, Japan

[3] Materials and Structures Laboratory, Institute of Integrated Research, Institute of Science Tokyo, 4259 Nagatsuta-cho, Midori-ku, Yokohama, Kanagawa 226-8501, Japan

[4] Department of Chemistry, Northwestern University, 2145 Sheridan Rd. Evanston, IL 60208-3113, United States

[5] Department of Materials Science, Graduate School of Engineering, Osaka Metropolitan University, 1-1, Gakuen-cho, Naka-ku, Sakai, Osaka 599-8531, Japan

[6] Department of Chemistry, Graduate School of Science, Kyoto University, Kitashirakawa Oiwake-cho, Sakyo, Kyoto 606-8502, Japan

E-mail:

tnishikubo@msl.titech.ac.jp (TN), krp@northwestern.edu (KRP), mazuma@msl.titech.ac.jp (MA)

Synthesis of Cu^{3+} containing $YBa_2Cu_3O_7$ from highly oxidized co-precipitation precursor

Experimental methods

・Preparation of precursor

Yttrium nitrate, barium nitrate and supper nitrate with stoichiometric metal ratios ware dissolved in dilute nitric acid. Co-precipitation and oxidation were carried out simultaneously by dropping above mentioned aqueous solution into an aqueous potassium carbonate / sodium hypochlorite mixture to obtain (ox)hydroxide gels. Using this precursor, $YBa_2Cu_3O_7$ (YBCO) were synthesized under HT condition.

・in-situ observation of the reaction process

in-situ observation of the reaction process was performed by synchrotron X-ray powder diffraction high-temperature using a high-temperature stage installed in BL13XU of SPring-8 with the energy of 35 keV.

・Characterization of the sample

To confirm the superconductivity, magnetic measurements were made in a SQUID magnetometer (Quantum Desing MPMS).

Results and discussions

It has been demonstrated that heating a precursor formed from broad peaks leads to the formation of an intermediate phase, followed by the formation of orthorhombic YBCO starting at around 850°C (Fig. S1). Magnetization measurements of YBCO obtained by heating the precursor at 900°C for 10 minutes revealed that T_c = 82 K, indicating that YBCO containing Cu^{3+} was successfully synthesized in a short time (Fig. S2). To synthesize YBCO that exhibits such superconductivity, it is typically necessary to sinter at high temperatures for a long period and then anneal at low temperatures. Since this method enabled the synthesis of orthorhombic YBCO that exhibits superconductivity directly at low temperatures and in a short time, we were able to confirm the usefulness of this approach.

Supplemental Figures

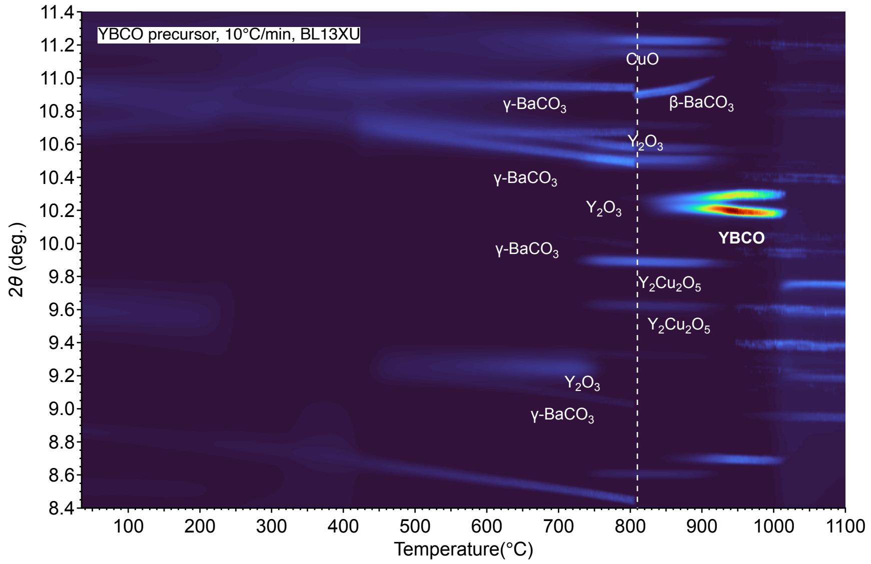

Figure S1 In situ observation of the synthesis process of YBCO using oxidized precursor

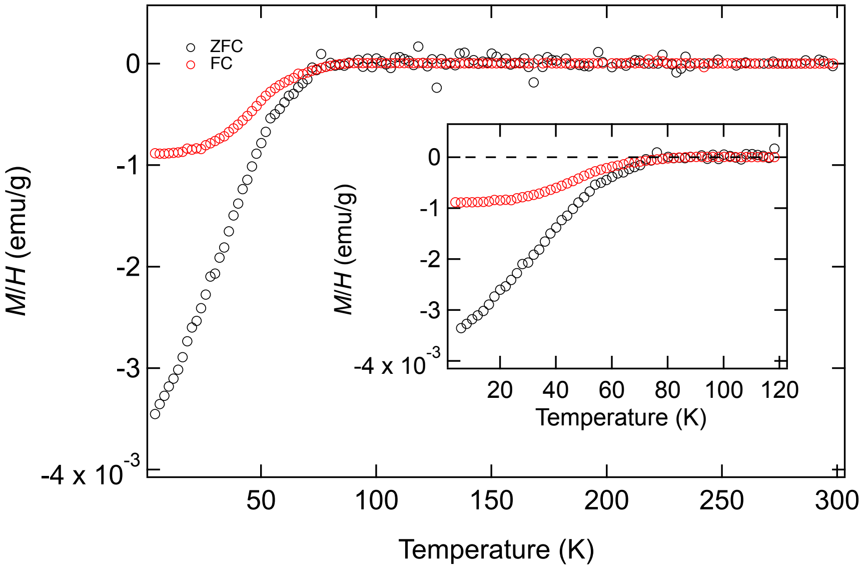

Figure S2 A magnetization measurement result for YBCO obtained after heating of oxidized precursor at 900°C for 10 minutes
